# Supplementary material for: Probing the upper band gap of atomic rhenium disulfide layers
Source: Light Sci Appl. 2018 Nov 28;7:98. doi: 10.1038/s41377-018-0100-3 (PMC6262017; doi:10.1038/s41377-018-0100-3)
Supplement: Supplementary file 1 — supplemental materials [file 41377_2018_100_MOESM1_ESM.docx]

Supporting Information

**Probing the Upper Band Gap of Atomic Rhenium Disulfide Layers**

Krishna P. Dhakal^1^, Hyunmin Kim^*,2^, Seonwoo Lee^1^, Youngjae Kim^3^, JaeDong Lee^*,3^

Jong-Hyun Ahn^*,1^

^1^School of Electrical and Electronic Engineering, Yonsei University, Seoul 03722, Republic of Korea.

^2^Companion Diagnostics & Medical Technology Research Group, DGIST, Daegu 42988, Republic of Korea.

^3^Department of Emerging Materials Science, DGIST, Daegu 42988, Republic of Korea.

*Authors to whom correspondence should be addressed to electronic addresses : hyunmin.kim@dgist.ac.kr, jdlee@dgist.ac.kr and ahnj@yonsei.ac.kr

**Table of Contents:**

1. Experimental and theoretical calculation detail
2. Identification of the layer numbers using PL and Raman spectroscopy
3. Thickness dependent modelling of the SHG and interference effect
4. Polarization dependent SHG and TSHG study
5. Exciton-exciton annihilation in the ReS_2_ crystal
6. Thickness dependent theoretical model for the decay dynamics
7. **Experimental and theoretical calculation detail**

**Raman, PL and absorption characterization:** The exfoliated ReS_2_ was first characterized using PL and Raman spectroscopies (Horiba Jovin Yvon, LabRAM Aramis) as well as AFM imaging (Park system, NX-10). The focal diameter for the PL and Raman measurements was approximately 500 nm. Reflected scattered light was collected through the objective lens used for illumination and guided to a 50-cm-long monochromator (equipped with a cooled CCD) via the free space. Diffraction gratings with 600 and 1,800 grooves mm^−1^ were used to collect PL and Raman spectra, respectively. A commercial un-polarized 532 nm solid-state cw laser equipped in a commercial confocal microscope platform was used for PL and Raman experiments with the intensities less than 300 μW (0.9-NA objective lens). Given power level of laser illumination, no physical damage or oxidation was expected to occur.^1,2^ And the absorption spectroscopy of the thin films were performed using a tungsten white light source already installed to the microscope system. The differential reflection is proportional to the absorption,^1,2^ hence, measures the layer dependent absorption in a broad energy range. In our experiment, we transferred the various exfoliated ReS_2_ layers to the transparent polydimethylsiloxane (PDMS) substrate.

**Nonlinear optical characterization:** More details of the nonlinear optical microscopy setup will be available in our previous reports.^2,3^ Briefly, an erbium-doped fiber oscillator (Spectra-Physics, Insight Deepsee Dual) was merged with a raster scanning (Olympus, Flouview 1000) microscopic platform (Olympus, IX 83) to create TSHG images of the exfoliated ReS_2_ flakes. A frequence-doubled (520 nm) fundamental ultrafast (80-MHz) pulse (1,040 nm, FWHM 220 fs) simultaneously drives the inside OPO (optical parametric oscillator) to emit a wavelength-tunable (from 680 nm to 1,300 nm) pulse train (FWHM 120 fs). A mechanical stage (Sigma-Koki, SGSP46-500) was adopted to control the time delay of the principle (probe) beam.

**Theoretical calculation:** We employed the first-principles electronic structure calculation performed on Vienna *ab-initio* simulation package (VASP) code with Perdew-Burke-Ernzerhof (PBE) generalized gradient approximation (GGA) exchange correlation functional^4-6^ and Monkhorst-Pack^7^ grid of **k**-points 9×9×1 mesh. Energy cutoff of 500 eV is adopted. Freestanding bilayer 1T’-ReS_2_ is calculated with Dudarev’s DFT+U^8^ correction of 5 eV for p orbitals of S atoms and substrate supported state is considered as compression of ~ -2 %.

1. **Identification of the layer numbers using PL and Raman spectroscopy**

We mechanically exfoliated the bulk ReS_2_ crystals (2D materials) onto 300 nm silicon dioxide (SiO_2_) coated silicon substrates and obtained different layer thickness crystals consisting of mono-, bi-, and tri-layer as shown in the optical micrograph (Figure S1a). Additionally, optical micrographes, Raman spectra, photoluminescence (PL) spectra and AFM images were taken to accurately assess the sample thickness. The Figure S1a illustrates the PL spectra obtained from the different layered ReS_2_ crystals, the thicknesses of which are labeled in the optical micrograph (top panel). All ReS_2_ films exhibit distinct measurable PL intensities in the range of 1.48–1.65 eV. The bottom panel of Figure S1a shows the thickness-dependent variation in PL peak positions and intensity. The peak energy for an isolated 1L was 1.66 eV, which became 1.55 eV in 2L; then, gradually decreased and eventually leveled off at around the 5L thickness (1.48 eV). On the other hand, the increasing PL with the increment in the layer number demonstrates a maximum in the bulk crystal. This type of thickness dependence of PL intensity in ReS_2_ crystals is markedly different from the Mo- and W-type transition metal dichalcogenide (TMD) crystals, where the PL intensity is the highest in 1L as a result of the weakening of the interlayer coupling and optimized band structure confinement at the K point.^1,2^ Our observations on thickness-dependent variation in the PL peak position and PL intensity are well matched with the previously reported results in the literature.^9-15^

Figure S1b shows the Raman spectra of the layers studied in Figure S1a. Multiple in-plane peaks observed near 150 cm^-1^ and 210 cm^-1^ as labeled in the Figure S1b indicates the highly asymmetrical structure of the ReS_2_ crystals. Figure S1c displays the Raman spectral peak and the frequency difference as a function of sample thickness. The Raman bands labelled as “i”, “iii” and “v” clearly demonstrates layer number dependency. Note that the frequency difference between peaks “iii” and “i” peaks (iii-i) decreases significantly with increasing of layer thickness and can become a good index to determine the layer thickness. Our Raman spectra are well matched with the previously reported results regarding the changes in the frequency differences and peak positions as a function of the layer numbers.^9-15^

**Figure S1.** Layer number dependent PL and Raman spectra of ReS_2_ crystals. a) Optical micrograph of 1L, 2L, 3L, 5L and bulk samples. PL spectra are also compared from each of the crystals. The PL peak position and intensity variation as a function of the layer number is plotted at the bottom. Well defined intensity enhancement and band gap reduction with increasing the layer thickness is observed. b) Corresponding normalized Raman spectra obtained from each of the layer thickness as shown in the figure. Each spectra are spaced for clarity. c) Frequencies for modes I, III, and V, labeled in b, have been plotted as a function of thickness (1L, 2L, 3L, 5L, and bulk) on the left vertical axis, while, the frequency difference between modes I and III on the right vertical axis. Raman and PL correlated study clearly reveals the layer thickness identification. d) Determination of the layer thickness using the optical micrograph (left, scale bar: 3 µm) and the AFM image (right, scale bar: 4 µm). The exact thickness of the region across the dash line in the AFM image was presented with the line profile.

**Figure S2.** Second harmonic generation (SHG) images of the ReS_2_ and MoS_2_ crystals. a) correlated optical and SHG micrographes of the region with various layer thickness (2L, 3L, 5L, 7-8L to bulk crystals) sample (scale bar: 5 µm). b) The optical and SHG image obtained from the 1L to 6L MoS_2_ crystals exfolidated on the Si/SiO_2_ substrate. The data plot (black ball) shows the experimental results. The red circle displays the results of a model (described in the text). The uncertainties in the measurement are indicated by the error bars.

1. **Thickness dependence modelling of the second harmonic generation (SHG)**

The distorted nature in the crystal symmetry of ReS_2_ can create a more complexity in the degree of interlayer coupling as well as symmetry variations than the II-IV TMDs. ^9-15^ In fact, the symmetry variation can be directly measured by comparing the layer thickness dependent second harmonic generation (SHG) intensity in a few numbered ReS_2_ crystals. However, the case gets more complicated as the layer thickness increases. As discussed in the main text, major contributing factors could be (a) an imperfection in the phase matching condition originated from various types of the stacking configurations and (b) an interference which even more modulates the optical signal in a bulk condition.^16,17^

The SHG intensity for the AA(A...) stacking of the *N* layer system can be modelled according to the electric-dipole approximation as^2,18^

$I\left( 2\omega\right)\propto N^{2}\left| \chi_{s}^{2} \right|^{2}$ (S1)

where *N* represents the number of layer and $\chi_{s}^{2}$ represents the effective surface second-order susceptibility tensor of the monolayer. The phase matching condition of the electric dipoles for the AA stacking (parallel dipole) and the dipole cancelation for the AB type stacking (anti-parallel dipole) are indicated in the schematic of the Figure S3a. This effect on the SHG emission is previously mentioned for the AA(A...) and AB(A...) type MoS_2_ in the reference 2 and reference 18. In conclusion, for the parallel dipole case (AAA.. stacking), the SHG signal increases quadratically with respect to the layer number as illustrated by the black ball in the Figure S3b. However, the SHG intensity in ReS_2_ increases more monotonically with the layer thickness increament, which is far below the predicted ~*N^2^* model as illustrated by the solid square in the Figure S3b. Such a deviation might be due to the imperfection in the phase matching condition, thus the net SH responses cannot sharply reflect the predicted *~N^2^* model. Various other factors can also modulate the quadratic dependence model (~*N*^2^), such as constructive interference by the neighboring dielectric layers, and reabsorption of SHG photons by the adjacent crystals.

To understand the observation more deeply, we performed the Fresnel calculation for different thickness ReS_2_ layers and obtained the field enhancement factor which is normally adopted for the study of the thickness dependent Raman and four-wave mixing intensity variation.^10-12^ The schematic in Figure S3d shows multiple reflections of light through the different layer structure. Here, we applied a similar model to understand the changes in the SHG intensity of ReS_2_ with different thicknesses. In this model, two photon absorption of the probe beam at 1040 nm (1.19 eV) and emission at the double of the probe frequency at 520 nm (2.38 eV) are separately considered. When the incident laser beam goes through the surfaces, it is absorbed partially by ReS_2_ layer and substantially by SiO_2_/Si substrate. And the laser beam also undergoes multiple reflections in ReS_2_ layer as well as in SiO_2_/Si substrate. So this multiply reflected beam also makes it possible to be absorbed in ReS_2_ layer. The net absorptions at a laser excitation wavelength of 1040 nm can be represented as *F*_ab_, expressed as,

$F_{\mathrm{ab}}=t_{1}\frac{\left[ 1+r_{2}r_{3}e^{-2i\beta_{2}} \right]e^{-i\beta_{x}}+\left[ r_{2}+r_{3}e^{-2i\beta_{2}} \right]e^{-i\left( 2\beta_{1}-\beta_{x} \right)}}{1+r_{2}r_{3}e^{-2i\beta_{2}}+\left( r_{2}+r_{3}e^{-2i\beta_{2}} \right)r_{1}e^{-2i\beta_{1}}}$

$F_{ab}=t_{1}\frac{\left[ 1+r_{2}r_{3}e^{-2i\beta_{2}} \right]e^{-i\beta_{x}}+\left[ r_{2}+r_{3}e^{-2i\beta_{2}} \right]e^{-i\left( 2\beta_{1}-\beta_{x} \right)}}{1+r_{2}r_{3}e^{-2i\beta_{2}}+\left( r_{2}+r_{3}e^{-2i\beta_{2}} \right)r_{1}e^{-2i\beta_{1}}}$ (S2)

where $t_{1}=\frac{{2n}_{0}}{n_{1}+n_{0}}$ is a Fresnel transmittance and $r_{1}=\frac{n_{0}-n_{1}}{n_{0}+n_{1}},r_{2}=\frac{n_{1}-n_{2}}{n_{1}+n_{2}}, r_{3}=\frac{n_{2}-n_{3}}{n_{2}+n_{3}}$ are the Fresnel reflectance coefficients for each interface between (1) oil (refractive index n_0_ = 1.51), (2) ReS_2_ (refractive index n_1_ = 4.3-0.001i), (3) SiO_2_ (refractive index n_2_ = 1.47) and (4) Si (refractive index n_3_ = 3.67-0.005i) (summarized in the table S1). $\beta_{x}=\frac{2\pi xn_{1}}{\lambda}$, $\beta_{1}=\frac{2\pi d_{1}n_{1}}{\lambda}, \beta_{2}=\frac{2\pi d_{2}n_{2}}{\lambda}$ are the phase change of the laser while passing through each layer where λ is the excitation wavelength and $x$ is the depth in ReS_2_ from which we get all interactions; d_1_ and d_2_ is the thickness of ReS_2_ and SiO_2_, respectively. Correspondingly, the net emission (EM) at a laser excitation wavelength of 520 nm can be represented as,

$F_{em}=t_{1}^{'}\frac{\left[ 1+r_{2}r_{3}e^{-2i\beta_{2}} \right]e^{-i\beta_{x}}+[r_{2}+r_{3}e^{-2i\beta_{2}}]e^{-i(2\beta_{1}-\beta_{x})}}{1+r_{2}r_{3}e^{-2i\beta_{2}}+(r_{2}+r_{3}e^{-2i\beta_{2}})r_{1}e^{-2i\beta_{1}}}$ (S3)

where $t_{1}^{'}=\frac{2n_{1}}{n_{1}+n_{0}}$ is the Fresnel transmittance. The parameters used in this calculation is n_0_ =1.51, n_1_ = 5.37-1.2i, n_2_ = 1.48, n_3_ = 4.2-0.05i. Then, the overall enhancement factor (F) is expressed as;

$F=\int_{0}^{d_{1}} \left| F_{ab} \right|^{4}\left| F_{em} \right|^{2}dx$ (S4)

The measured second harmonic generation intensity ($I_{shg})$ is given by $I_{shg}= I_{parmeter}\cdot F$ where $I_{parmeter}$ is the fitting parameter for the calculation.

We found that the interference effect positively aids, to the SHG response with a maximum contribution near the 10 nm thickness and then decreased drastically, however the maximum intensity predicted by the interference effect is much smaller than the experimentally observed SHG intensity. Compared with 1L, the SHG for ~13 L was enhanced by 20 times while the interference effect provide just a 4.5 times higher one (Figure S3b). This clearly indicates that the residual SHG enhancement depends on the phase matching condition of the SH dipoles induced by the stacking orientations among the stacked layers. Hence, the general expression for the thickness dependent SHG model of the atomically thin ReS_2_ could be expressed as,

$I\left( 2\omega\right)\propto N^{2}\left| \chi_{s}^{2} \right|^{2}\left| F \right|^{2}f(\phi)$ (S5)

where $\left|F\right|^{2}$is the electric field enhancement due to interference effects owing to the various dielectric layers; *f (*$\varphi$) is the phase function which depends on the angle between the input laser polarization and the initial crystallographic orientation ($\varphi$).

Moreover, a linearly increased absorption coefficient in the SH photon energy (~2.38 eV), particularly as the layer number increases^12^(Figure S4), also affects the observed SH emission property possibly due to re-absorption of SH photons with increasing thickness. The measured absorption spectra for different layer clearly reveals the increasing tendency of the absorption, predominantly in the SH photon energy range (supporting information Figure S4). Also, it has been already known that any misalignment of the parallel SH dipoles by controlling the stacking orientation angles of the stacked MoS_2_ crystal leads to a significantly weak SHG signal.^2^ Therefore, the weak ~*N^2^* dependent SH intensity in the ReS_2_ crystal (Figure S3b) compared to the prior result of 3R-like MoS_2_^2,18^ strongly suggests the possible misalignment of the orientation angles among all stacked layers. And, this consequences a weak interlayer coupling of multilayer ReS_2_ crystal at which the chance of the diverse range of stacking misconfigurations of each stacked layer cannot be ignored. For a crystal with a thickness more than 16 nm, a drastic reduction in the SH response was reached to an order of loss for a bulk limit, which is consistent with the observations from the other class of bulk TMDs.^18,19^

**Figure S3.** Thickness dependent SH intensity and interferometric absorption of the layered ReS_2_ crystals. a) Schematic of the atomic arrangement of the 1L (top view) and 2L (side view) ReS_2_ and that of MoS_2_ for comparison. The direction pointed out by the arrow represents the direction of the SH polarization vector. The parallel arrangement creates a constructive SH interference condition while an anti-parallel configuration cancel out that effect as illustrated in the 2L-exfoliated MoS_2_. The net SH emission process in the ReS_2_ crystal can be understood as the combined contribution from b) the electric dipole approximation (black ball, ~*N*^2^) and the interference effect (black triangles). The red circle in the Figure S3b is the product of the interference model and *N^0.6^* dependence with layer numbers. The factor of ~0.6 was obtained by fitting the electric dipole approximation of the *~N^2^* model of MoS_2_ like crystal at the maximum experimental condition. The inset represents the SHG spectrum for the probe wavelength of 1040 nm. c) The schematic (at the top) represents the light absorption occuring in the multiple layers. We considered the different refractive index values and obtained the field enhancement due to Fresnel calculation. A comparison of the SH field enhancement due to light incidence through air and oil medium is shown in the Figure S3c.

**Table S1**. Refractive index values for the different materials and wavelengthes of incident beam.

**Figure S4.** Layer thickness dependent absorption spectra of ReS_2_ crystals taken from the sample deposited on the transparent substrate (Polydimethylsiloxane, PDMS). a) Optical micrographs of exfoliated samples are shown at the top left and the corresponding scale bar is 5µm. b) PL spectra displayed at the top right, demonstrated the well defined intensity enhancement and band gap reduction with the layer thickness increase. c) Corresponding reflection spectra obtained from each of the layer thickness are shown in the bottom of the figure. These reflection spectra are directly proportional to the absorption spectra^1^ especially with a strong absorption in the high energy region and an increment linearly with the increase of the layer thickness in a wide energy range (1.35-3 eV). The gradual red shift of the exciton around the 1.57 eV with the thickness increment is highlighted in the inset of the graph. Our result is similar to the previously reported results of the ReS_2_ crystal.^12^

1. **Polarization dependent SHG and TSHG study**

**(a) Polarization property of the SHG**

The polarization dependent SHG behavior determines the orientation of the crystallographic axis of any crystal and help to identify the symmetry point group. We measured the polarization dependence of the SHG signal with and without excitation of the pump pulses correspondent to the excitonic resonance condition. Polar plots shown in the Figure S5 and S6 directly compared the SHG anisotropy with probe only and pump plus probe excitation, respectively for the 2L and 8~9L samples as a function of the sample rotation angle. Here, the analyzer and the polarization of the excitation beam were adjusted parallel to each other. The anisotropic SHG responses of the 2L ReS_2_ crystal demonstrates a butterfly-like pattern in Figure S5a (left plot). In contrast, the polarization dependence of 8~9L displays a squeezed two-lobe circular pattern. The observed SHG pattern from the ReS_2_ crystal is consistent with that of the C_s_ point group such as 1T′ phase MoTe_2_ crystal. The incident electric field (*I_ω_*) generates a second harmonic signal (*I_2ω_*) along the directions for parallel and crossed polarizations, respectively. Then, our SHG signal is expressed as $I(2\omega)\propto\left|\alpha\cos^{3}\theta+\beta\cos\theta\sin^{2}\theta\right|^{2}$since the beam and analyzer directions are parallel.^20^ A fitting process provides the fitting constants of the polarization dependent SHG signals as, α = 0.5 and β = 2.25 for 1L; α = 0.85 and β = 1.90 for 8~9L. We believe that any deviations from the thin layer may occur due to substrate induced strain effect or other possible structural imperfection.

**(b) Polarization dependence of TSHG**

The contributions to the second-harmonic (SH) field (*E*_2_*_ω_*) of the probe (*E_ω_*) in the presence of the pump (*E*_p_) electric field excitation can be defined similarly to the previously described manner for MoS_2_^3^ as,

$E^{2\omega}=E_{\omega}^{2\omega}+E_{p}^{2\omega}\propto\chi_{ReS_{2}}^{2}E^{\omega}E^{\omega}+\chi_{ReS_{2}}^{3}E^{\omega}E^{\omega}E_{p}$ (S6)

where $\chi_{ReS_{2}}^{2}$ and $\chi_{ReS_{2}}^{3}$ represents the second and third order of susceptibility of the ReS_2_. Therefore, the experimentally observable SHG intensity can be expressed as follows:

$I^{2\omega}=\left| E_{\omega}^{2\omega}+E_{p}^{2\omega} \right|^{2}=\alpha\left| \chi_{ReS_{2}}^{2} \right|^{2}\left| I^{\omega} \right|^{2}+\beta{\chi_{ReS_{2}}^{2}\chi}_{ReS_{2}}^{3}\left| I^{\omega} \right|^{2}E_{p}+\gamma\left| \chi_{ReS_{2}}^{3} \right|^{2}I^{p}\left| I^{\omega} \right|^{2}$ (S7)

where $\beta$ and $\gamma$ are the constants that depend on the geometry, $I^{\omega}$ and $I^{p}$ are the incident laser field intensities of the probe and pump beams, respectively.

Thus, the intensity of the transient second harmonic generation (TSHG) could be expressed as,

$\Delta I^{2\omega}=I^{2\omega}-I_{0}^{2\omega}=$ $\beta{\chi_{ReS_{2}}^{2}\chi}_{ReS_{2}}^{3}\left| I^{\omega} \right|^{2}E_{p}+\gamma\left| \chi_{ReS_{2}}^{3} \right|^{2}I^{p}\left| I^{\omega} \right|^{2}$ (S8)

On the other hand, the phase of the equation S8 with regards to the 1T’ distorted crystalline is,

$$\Delta I^{2\omega}=k\left[ \cos\left( \theta-\theta_{11} \right)\left\{ \alpha{cos}^{3} \left( \theta-\theta_{12} \right)+\beta\cos\left( \theta-\theta_{12} \right){sin}^{2} \left( \theta-\theta_{12} \right) \right\} \right]+r\left[ \cos^{2} \left( \theta-\theta_{21} \right)\left\{ \alpha{cos}^{3} \left( \theta-\theta_{22} \right)+\beta\cos\left( \theta-\theta_{22} \right){sin}^{2} \left( \theta-\theta_{22} \right) \right\} \right]$$

(S9)

where *k* and *r* are constants; θ is the angle between the polarization of the probe light and the sample orienration having a maximum SHG signal (b axis). θ_11_, θ_12_, θ_21_, and θ_22_ are phase differences between E_p_ (pump) and probe beam; probe and probe beam; I_p_ (pump) and probe beam; probe and probe beam, respectively. Here, the equation S9 can be shortened because there is no phase factor (θ_xx_=0) when pump and probe fields are parallel,

$\Delta I^{2\omega}\propto(kcos\theta+r{cos}^{2}\theta)\left( \alpha{cos}^{3} \theta+\beta\cos\theta{sin}^{2} \theta\right)\propto(kcos\theta+r{cos}^{2}\theta)I(2\omega)$

(S10)

In the presence of the pump beam, the absolute value of the SHG signal of the ReS_2_ crystal has decreased. The green ball in the polar plots (Figure S5 and S6) displays the SHG tendency in the presence of the pump beam where no observable phase shift is acknowledged. We also monitored the polarization-sensitive behavior of the SHG signal obtained as the difference between the usage of the pump beam (ΔI, TSHG). Blue balls in the polar plots in Figure S5 and S6 are corresponding to TSHG.

**Figure S5.** Anisotropic SHG and TSHG property of the multi-layer ReS_2_. a) Polarization dependent SHG and TSHG intensities with regards to the sample rotation of 8-9 L ReS_2_ crystal. The optical image of the sample with the direction of the crystalline axis is shown at the right. The polar plots shows the polarization dependent variation in the SHG intensity of probe beam (1.19 eV) in the absense (black ball) and presence (green ball) of pump beam (1.57 eV). The difference represents the TSHG and plotted with blue ball, indicating TSHG has a small phase shift compared with SHG. b) SHG images as a function of the sample rotation in the absence and presence of pump excitation (scale bar: 5 µm). The double sided arrow indicates the direction of the two parallel excitation beam polarizations. (c, d) PL and Raman spectra of the analyzed crystal confirmed the layer thicknesses of ReS_2_ crystals.

**Figure S6**. Anisotropic SHG and TSHG property of the bi- layer ReS_2_. a) Optical micrograph, Raman, and PL spectra of the 2L-ReS_2_ crystal confirm the layer thickness. b) Polarization dependent SHG in the presence (black ball) and absence (green ball) of pump beam and the difference (TSHG, blue ball) intensity with regards to the sample rotation from the typical 2L crystal. Solid red lines fit the theoretical model to the experimental data as explained in the main text (b). (c) SHG images show the sample rotations in the present and absence of the pump excitation (scale bar: 5 µm). The double sided arrow indicates the direction of the two parallel excited beam polarizations.

**Figure S7.** Polarization dependent SHG of thick (~13-16 L) ReS_2_ crystals with regards to the sample rotation. SHG images in the presence and absence of pump excitation are indicated at the bottom panel of the figure (scale bar: 2 µm). The double sided arrow indicates the direction of the two parallel excited beam polarizations. Data were analyzed as for the region with the asterisk.

**Figure S8.** Pump fluence dependent TSHG images as a function of the probe delay time. The valley of the TSHG signal in the decay region has deepened with the higher pump fluence illumination (scale bar: 2 µm). The numbers of the pump fluence, pump energy, and probe energy are noted in the figures.

1. **Exciton-exciton annihilation in the ReS_2_ crystal**

To get a clearer intuition regarding the pump fluence dependent TSHG tendency as addressed in the main Figure 3a, we first modelled the decay-to-rise kinetics according to the exciton-exciton annihilation process previously reported as,^21^

$N_{x}\left( t \right)=\frac{N_{0}}{1+K_{A}N_{0}t}$ (S11)

where *K*_A_ represents the rate constant for the annihilation process, *N_0_* is the initial photogenerated exciton population. We approximately estimated the fluences dependent number density by correlating our pump power level with the previously measured absorption coefficient; the pump fluence varies from 0.6 to 8 mJ cm^-2^ and the absorption coefficient is 8×10^6^ m^-1^ (bulk value).^22^ Additionally, by assuming that each absorbed pump photon generates one electron–hole pair, we could estimate the surface carrier density injected (*N_0_*) at the center of the pump spot (~300 nm) at each power level. Then, the transient exciton population was obtained by fitting the K value in the above equation with regards to the normalized TSHG spectra, as is summerized in the Figure S9. The average value of the K was estimated as 0.7 cm^2^/s, indicating a strong exciton-exciton annihilation process happens in the ReS_2_ crystals.

**Figure S9.** TSHG profiles obtained from a few layered ReS_2_ at room temperature measured at two pump fluences of a) 0.6 and b) 8 mJ/cm^2^. The right axis indicates the calculated numbers of the photogenerated excitons. The solid lines represent a global fit according to the exciton-exciton annihilation model in the text.

**Figure S10.** Defect related PL modulation as a result of the increasing carrier population on the ReS_2_ crystal. a) Confocal PL spectra of the ~13 nm thick crystals obtained with the excitation of the 2.33 eV focused laser beam at various power as mentioned in the figure. Each PL spectra were fitted by Gaussian curve and the change in the intensity, peak width, and peak positions have been analyzed in the right panel. The PL spectra were taken from the same layers used for the pump power dependent TSHG in Figure 4b. b) Peak positions, c) Peak intensity, and the full width at half maximum of the corresponding PL band were measured at the different fluence levels. The spectral shape of the PL spectra have been broadened with higher laser power towards the lower energy, in agreement with the observation of the defect state trapping as a result of the increasing carrier population generation with the bombardment of excitation photons of high flux density. The nonlinear tendency PL intensity and PL band width variation with increasing the excitation power also reflects the defect mediated e-h recombination in the ReS_2_ as of conventional TMDs.^23^

1. **Thickness dependent theoretical model for the decay dynamics**

In order to discriminate the surface and bulk recombination of multi-layer ReS_2_ films, we adopted a model assuming a quantized presence of the electrons and holes in the solids established in the MoS_2_ case.^25, 26^  In this model, the lifetime (τ) of the carriers in a few-layer (N ≥ 2) sample was defined as,

$\frac{1}{\tau}=\frac{{sin}^{2}\left( \frac{\pi k}{2} \right)+\frac{2}{1+N}{sin}^{2}(\frac{\pi k}{1+N})}{2\tau_{A}}+\frac{{1+0.5[sin}^{2}\left( \frac{\pi k}{2} \right)+\frac{2}{1+N}{sin}^{2}(\frac{\pi k}{1+N})]}{\tau_{B}}$ (S12)

where *N* is the total number of layers until the k-th particular layer, $\tau_{A}$represents the fast exciton-exciton recombination time and $\tau_{B}$ is a slow bulk-related recombination time. We fitted the values of $\tau_{A}$ and $\tau_{B}$as 50 ps and 125 ps, respectively.

**Figure S11.** Decay-to-rise SHG tendency of the ReS_2_ crystals with the illumination of the pump beam at 1.57 eV and the probe beam at 1.19 eV. The trace of the dashes line in the sequential TSHG images clearly guides the tendency of the decay-to-rise as a function of the probe delay time.

**Figure S12.** Thickness dependent decay constant of the ReS_2_ crystals. Bi-exponential fitting to explore the fast (blue circle) and slow (red ball) components of the decay constant from the different layer thicknesses ReS_2_ crystals shown in Figure 3b. The result indicates that the total carrier life time is mainly governed by the slow decay with increasing layer thickness.

**Figure S13.** Comparision of the anisotropic TSHG property of the 6L ReS_2_ crystal measured at two different pump probe conditions as mentioned in the respective plot. An optical contrast of the sample region is also shown as an inset. Observed result clearly shows the absence of the phase shift between TSHG signal obtained from different pump probe conditions. Corresponding TSHG images with regards to the sample rotation angle are shown in the left.

**References**

# Dhakal KP, Duong DL, Lee J, Nam H, Kim M *et al.* Confocal absorption spectral imaging of MoS_2_: optical transitions depending on the atomic thickness of intrinsic and chemically doped MoS_2_*.* *Nanoscale* 2014; 6: 13028-13035.

# Shinde SM, Dhakal KP, Chen X, Yun WS, Lee JD *et al*. Stacking-controllable interlayer coupling and symmetric configuration of multilayered MoS_2_, *NPG Asia Mater* 2018; 10: e468.

# [Jang H](https://www.ncbi.nlm.nih.gov/pubmed/?term=Jang%20H%5BAuthor%5D&cauthor=true&cauthor_uid=29436068), [Dhakal KP](https://www.ncbi.nlm.nih.gov/pubmed/?term=Dhakal%20KP%5BAuthor%5D&cauthor=true&cauthor_uid=29436068), [Joo KI](https://www.ncbi.nlm.nih.gov/pubmed/?term=Joo%20KI%5BAuthor%5D&cauthor=true&cauthor_uid=29436068), [Yun WS](https://www.ncbi.nlm.nih.gov/pubmed/?term=Yun%20WS%5BAuthor%5D&cauthor=true&cauthor_uid=29436068), [Shinde SM](https://www.ncbi.nlm.nih.gov/pubmed/?term=Shinde%20SM%5BAuthor%5D&cauthor=true&cauthor_uid=29436068) *et al*. Transient SHG Imaging on Ultrafast Carrier Dynamics of MoS_2_ Nanosheets. [*Adv Mater*](https://www.ncbi.nlm.nih.gov/pubmed/29436068)2018;30:1705190.

# Kresse G, Furthmüller J. Efficiency of ab-initio Total Energy Calculations for Metals and Semiconductors Using a Plane-Wave Basis Set. *Comp Mater* Sci 1996; 6: 15-50.

# Perdew JP, Burke K, Ernzerhof M. Generalized Gradient Approximation Made Simple. *Phys Rev Lett* 1996; 77: 3865-3868.

# Kohn W, Sham LJ. Self-Consistent Equations Including Exchange and Correlation Effects. *Phys Rev* 1965; 140: A1133-A1138.

# Monkhorst HJ, Pack JD. Special Points for Brillouin-Zone Integrations. *Phys Rev* B 1976; 13: 5188-5192.

# Dudarev SL, Botton GA, Savrasov SY, Humphreys CJ, Sutton AP. Electron-Energy-Loss Spectra and the Structural Stability of Nickel Oxide: An LSDA+ U Study. *Phys Rev B* 1998; 57: 1505.

# Tongay S, Sahin H, Ko C, Luce A, Fan W *et al.* Monolayer behavior in bulk ReS_2_ due to electronic and vibrational decoupling. *Nat Commun* 2014; 5: 3252.

# Chenet DA, Aslan OB, Huang PY, Fan C, van der Zande AM *et al*. In-Plane Anisotropy in Mono- and Few-Layer ReS_2_ Probed by Raman Spectroscopy and Scanning Transmission Electron Microscopy. *Nano Lett* 2015; 15: 5667.

# Feng Y, Zhou W, Wang Y, Zhou J, Liu E *et al.* Raman vibrational spectra of bulk to monolayer ReS_2_ with lower symmetry. *Phys Rev B* 2015; 92: 054110.

# Aslan OB, Chenet DA, van der Zande AM, Hone JC, Heinz TF *et al.* Linearly Polarized Excitons in Single- and Few-Layer ReS_2_ Crystals. *ACS Photonics* 2016; 3: 96-101.

# He R, Yan J-A, Yin Z, Ye Z, Ye G *et al.* Coupling and Stacking Order of ReS_2_ Atomic Layers Revealed by Ultralow-Frequency Raman Spectroscopy *Nano Lett* 2016; 16: 1404-1409.

# Qiao XF, Wu JB, Zhou L, Qiao J, Shi W *et al.* Polytypism and unexpected strong interlayer coupling in two-dimensional layered ReS_2_ *Nanoscale* 2016; 8: 8324-8332.

# Cui Q, Muniz RA, Sipe JE, Zhao H *et al.* Strong and anisotropic third-harmonic generation in monolayer and multilayer ReS_2_ *Phy. Rev. B: Condens. Matter Mater. Phys*. 2017; 95: 165406.

1. Lee S, Kim K, Dhakal KP, Kim H, Yun, WS *et al.* Thickness-Dependent Phonon Renormalization and Enhanced Raman Scattering in Ultrathin Silicon Nanomembranes. *Nano Lett* 2017; **17**: 7744-7750.
2. Hendry E, Hale PJ, Moger J, Savchenko AK *et al*. Coherent Nonlinear Optical Response of Graphene. *Phy Rev Lett* 2010; **105**: 097401.

# Zhao M, Ye Z, Suzuki R, Ye Y, Zhu H *et al.* Atomically phase-matched second-harmonic generation in a 2D crystal, *Light Sci App* 2016; 5: e16131.

# Li Y, Rao Y, Mak KF, You Y, Wang S *et al.* Probing symmetry properties of few-layer MoS_2_ and h-BN by optical second-harmonic generation*. Nano Lett* 2013; 13: 3329-3333.

1. Beams R, Cancado LG, Krylyuk S, Kalish I, Kalanyan B *et al.* Characterization of Few-Layer 1T′ MoTe_2_ by Polarization-Resolved Second Harmonic Generation and Raman Scattering. ACS Nano 2016; **10**: 9626-9636.

# Sun D, Rao Y, Reider GA, Chen G, Brezin YL *et al.* Observation of Rapid Exciton-Exciton Annihilation in Monolayer Molybdenum Disulfide, *Nano Lett* 2014; 14: 5625-5629.

# Cui Q, He J, Bellus MZ, Mirzokarimov M *et al.* Transient Absorption Measurements on Anisotropic Monolayer ReS_2_, small 2015; 11: 5565–5571.

# Tongay S, Suh J, Ataca C, Fan W, Luce A *et al*. Defects Activated Photoluminescence in Two-Dimensional Semiconductors: Interplay between Bound, Charged, and Free Excitons. *Sci Rep* 2013; 3: 2657.

# Friemelt K, Kulikova L, Kulyuk L, Siminel A, Arushanov E *et al.* Optical and Photoelectrical Properties of ReS_2_ Single Crystals. *J Appl Phys* 1996; 79: 9268.

# Wang H, Zhang C, Rana F. Ultrafast Dynamics of Defect-Assisted Electron-Hole Recombination in Monolayer MoS_2_, *Nano Lett* 2015; 15: 339-345.

# Wang H, Zhang C, Rana F. Life times of Photoexcited Carriers in Few-Layer Transition Metal Dichalcogenide MoS_2_, *Nano Lett* 2015; 15, 8204-8210.
