# Supplementary material for: Probing the upper band gap of atomic rhenium disulfide layers
Source: Light Sci Appl. 2018 Nov 28;7:98. doi: 10.1038/s41377-018-0100-3 (PMC6262017; doi:10.1038/s41377-018-0100-3)
Supplement: Supplementary file 2 — manuscript_highlighted for reviewer [file 41377_2018_100_MOESM2_ESM.doc]

Probing the Upper Band Gap of Atomic Rhenium Disulfide Layers

Krishna P. Dhakal1, Hyunmin Kim2,*, Seonwoo Lee1, Youngjae Kim3, JaeDong Lee*,3 
Jong-Hyun Ahn*,1

1School of Electrical and Electronic Engineering, Yonsei University, Seoul 03722, Republic of Korea.
2Companion Diagnostics & Medical Technology Research Group, DGIST, Daegu 42988, Republic of Korea.
3Department of Emerging Materials Science, DGIST, Daegu 42988, Republic of Korea.


Abstract
 Here, we investigate the ultrafast carrier dynamics and electronic states of exfoliated ReS2 films using time-resolved second harmonic generation (TSHG) microcopy and density functional theory (DFT) calculations. The second harmonic generation (SHG) of layers with various thicknesses is probed using a 1.19-eV beam. Up to ~13 nm, a gradual increment is observed, followed by a decrease caused by bulk interferometric light absorption. The addition of a pump pulse tuned to the exciton band gap (1.57 eV) creates a decay-to-rise TSHG profile as a function of the probe delay. The power and thickness dependencies indicate that the electron–hole recombination is mediated by defects and surfaces. The two photon absorptions of 2.38 eV in the excited state that are induced by pumping from 1.57 to 1.72 eV are restricted because these transitions highly correlate with the forbidden d–d intrasubshell orbital transitions. However, the combined usage of a frequency-doubled pump (2.38 eV) with wavelength-variant SHG probes (2.60-2.82 eV) allows us to vividly monitor the variations in TSHG profiles from decay-to-rise to rise-to-decay, which imply the existence of an additional electron absorption state (s-orbital) at an approximate distance of 5.05 eV from the highest occupied molecular orbital states. This observation was critically examined by considering the allowance of each electronic transition and a small upper band gap (~0.5 eV) using modified DFT calculations. 

Keywords: ReS2, SHG, TSHG, carrier dynamics, intraconduction band transition

Introduction
Rhenium disulfide (ReS2) is a group-VII transition metal dichalcogenide (TMD) material that exhibits considerable potential for photovoltaic applications due to its remarkable optoelectronic properties1−9 such as a direct band gap and a large work function.8, 9 ReS2 is characterized as containing a rich optical and electronic structural diversity due to the stable distorted triclinic 1T crystalline phase owing to the in-plane anisotropic excitons and phonons2-7 compared with the isotropic 2H crystal of group-VI TMDs, such as MoS2 and WSe2.10−19 Thus, increased attention has been devoted to investigating the resolution of the peculiar electronic and optical properties of the atomic layers of ReS2; the density of the electronic valence band states was directly measured using the angle-resolved photoemission spectroscopy (ARPES) technique,20 whereas the formation of birefringent excitons was identified using polarization fluorescence microscopy4. Additionally, the excitonic lifetime and diffusion coefficient were investigated using the pump–probe optical transient absorption technique,21 which also explained the blueshift in absorption in the presence of a highly intense pump fluence induced by the optical Stark effect.22 Even today, a deeper understanding of the static and dynamic features of the optoelectronic properties of ReS2 systems is required to develop practical device applications.
 The second harmonic generation (SHG) of atomically thin group-VI TMDs has been extensively studied because of the high second order susceptibility (χ2) and crystalline structural selectivity. Recently, confocal SHG microscopy was used to identify the relative orientations of the underlying layers in multilayer TMD crystals,12,17 which were not completely understood in the case of ReS2 due to the poor SHG conversion efficiency associated with the complex crystalline symmetry. In principle, the 1L ReS2 crystal (Ci point group) does not exhibit a noncentrosymmetric structure,3 in which the asymmetric inversion center is only associated with Au, whereas the Ag harmonic oscillation modes are symmetric. Thus, the polarization-dependent SHG signals of ReS2 crystals are not caused by the dependency of Au on the increment in the layer number but rather by the stacking alignment-induced broken inversion center, which is similar to that observed in 3R-like MoS2 crystals.12, 17 Furthermore, the aforementioned SHG “probe” could be utilized to create a time-resolved SHG (TSHG) spectroscopic condition characterized by the synchronized illumination of the pump pulse beam, which features the ultrafast carrier dynamics of atomically thin MoS2 even without an SHG contrast (i.e., for even-number MoS2 layers).18,23 In TSHG spectroscopy, the probing action of SHG is analogous to single-photon transient absorption, which is observed at the energy of the SHG of the probe photon.22,23 More specifically, Jang et al. 18 employed TSHG microspectroscopy to visualize the structure-dependent electron and phonon behavior with a spatial resolution of ~300 nm. In the excited state, absorption occurred at ~4.20 eV (by combining the 1.82 eV pump and the 2.38 eV SHG probe) from the valence band of MoS2; this was not observed for WSe2 above 4.03 eV (by combining the 1.65 eV pump and the 2.38 eV SHG probe). Additionally, Lindenberg et al.23 reported that an electronic transition to a distance of 6.02 eV from the ground state (by combining the 3.61 eV pump and the 2.41 eV SHG probe) of the monolayered MoS2 is allowed using TSHG spectroscopy. The electronic transition is not allowed at 7.76 eV (by combining the 4.66 eV pump and the 3.10 eV SHG probe). Given the aforementioned results and background, the time- and pump/probe energy-dependent SHG of various ReS2 systems should be investigated in more detail.
In this study, we investigated the ultrafast intraconduction band transitions of ReS2 crystals using pump–probe TSHG microscopy and DFT calculations. First, it was shown that the layer thickness-dependent SHG by 1.19-eV optical pulses quantitatively increases as a function of the layer number up to a thickness of ~13 nm and then gradually decreases, exhibiting a weak signal for the bulk counterpart. The polarization tendency of ReS2 also differed from that of the renowned molybdenum- and tungsten-based dichalcogenides, in which the SHG emission alters with odd or even layer numbers, rather resembling that of MoTe2. The addition of an excitonic wavelength (1.57 eV) pump caused a time-dependent decay-to-rise trend of the transient SHG signal originating from the ground state depletion (GSD) due to the lack of a permitted excited electron state at ~2.38 eV in the lowest unoccupied molecular orbital (LUMO) of the conduction band. A highly limited stimulated emission effect is expected due to the difference between the energy of the probe photon (1.19 eV) and that of the excitonic resonance (1.57 eV). The pump fluence dependence of the inverse lifetimes of excitons confirmed that the ultrafast carrier relaxation of ReS2 is highly affected by the crystalline defects. Further, the carrier lifetime change as a function of the number of layers matches that of the surface defect-quantized model well, indicating that the defect that is highly localized on the surface plays a major role in the recombination of the electron and hole in the ReS2 substrate. Direct optical pumping at a high energy (2.38 eV) demonstrated a transition of the TSHG profiles from decay-to-rise (2.60 eV) to rise-to-decay (2.82 eV), indicating possible absorption at the gamma point of the Brillion zone in the excited state at a distance of ~5.05 eV from the highest occupied molecular orbital (HOMO) of the valence band.
Results
	The schematic depicted in Figure 1a illustrates the microscopic setup to conduct the SHG study of the ReS2 crystal. More details about the TSHG optical microscopy setup were reported previously (refer to the Section 1, Supporting Information).18 A dual-mode Er-doped fiber laser system (Spectra-Physics, Insight Deepsee Dual) was used as a laser pumping oscillator (80 MHz) for a confocal galvanometric scanning (Olympus, Fluoview 1000) microscope (Olympus, IX 83) to create the SHG images of the exfoliated ReS2 flakes. One dichroic mirror (DMSP1000R, Thorlabs) was used to spatially overlap the wavelength-variable (120 fs, 680–1300 nm) and 220 fs, 1040 nm pulse beams; the other mirror (RDM690, Olympus) was used to extract the SHG signal from the incident photons. The oil immersion lens (UPlanFLN, Olympus) in our microscopic system allowed us to enhance the signal-to-noise ratio (1.35 NA) and simplified the heat dissipation from the sample. We mechanically exfoliated the ReS2 crystals (2D material) that were tethered on 300-nm silicon dioxide (SiO2)-coated silicon substrates. Figure 1b depicts SHG images representative of the mono-, bi-, and trilayer along with bulk systems and the experimental and simulated intensity profiles as a function of the sample thickness with ~10 mW illumination at the sample. The optical micrographs corresponding to the SHG images are depicted in the inset of the lower intensity profile. The exact layer thickness of the respective crystal was confirmed using optical contrast, photoluminescence (PL) spectroscopy, Raman spectroscopy, and atomic force microscopy (Figure S1 in Section 2, Supporting Information). A weak SH signal from the monolayer was observed at the substrate interface, while the SH signals from the bilayer and the other thicker layer were strongly enhanced in contrast to the 2H MoS2, as illustrated in the mapping images of the SH (Figure 1b and Figure S2, Supporting Information). In principle, the SHG output is maximized if the input laser and the output SHG signal in a system with noncentrosymmetry have identical polarization parallel to the crystalline surface. We rotated the sample by fixing the analyzer and polarizer in a direction parallel to the linearly polarized light and further determined the maximum SHG contrast. The plot in the lower part of Figure 1b displays the maximum SHG intensity produced by the various layers of ReS2 crystals. Note that the monolayer of ReS2 itself is not a perfectly noncentrosymmetric material like other well-known TMD monolayers, such as 1L MoS2. In principle, the 1L ReS2 crystal belongs to the Ci point group, which does not exhibit a noncentrosymmetric structure3. However, in the presence of a substrate interface, a broken inversion symmetric center could still exist in the centrosymmetric atomic layers,24 allowing for a weak SHG response of 1L ReS2. The SHG intensity increases with the layer number in systems with more than one layer, maximizes at a layer thickness of ~13 nm, and then decreases.
The SHG intensity in the Nth layer of an AAA-type 3R MoS2 system increases quadratically as a function of the layer number (Figure S3, Supporting Information).12, 17 However, various factors can modulate the quadratic dependence model (~N2), such as the constructive interference by the neighboring dielectric layers, reabsorption of SHG photons by the adjacent crystals, and net variation in the SH dipole moment, which is a function of the stacking sequence with increasing sample thickness (Figure S3 and S4 in Section 3, Supporting Information). Considering all these parameters, the observed layer-dependent SHG tendency of a low-symmetry crystal, such as ReS2, can be modeled as
 
  			 ,..........................................(1)

where is the electric field enhancement induced by the SiO2 layer and f() is the phase function dependent on the angle between the input laser polarization and the initial crystallographic orientation. Here, the power factor (n) of N was maintained at 0.6. 
To characterize the anisotropic SHG behavior of the ReS2 crystals, we measured the polarization dependency of the SHG signal. Figure 1c depicts the optical images of the 1L and 8–9L samples, which were used to monitor the polarization dependence; the SHG intensities were plotted (Figure 1d and e) as a function of the sample rotation angle, where the analyzer and polarization of the beam (1.19 eV) were adjusted parallel to each other. More details of the anisotropic SHG are provided in Section 4 of the Supporting Information, Figure S5-S7. The anisotropic SHG responses of the 1L ReS2 crystal represent a butterfly-like pattern in the corresponding polar plots (Figure 1d). However, the polarization dependence of the 8–9L samples exhibited a more tweaked two-lobe circular pattern. The SHG patterns for the ReS2 crystal were consistent with that of the nonlinear optical properties of the Cs point group, which was analogous to the 1Tʹ phase MoTe2 crystal.25 Additionally, a few TEM studies revealed the appearance of in-plane diamond-like Re in the ReS2 1T' phase.1,2,26,27 In this geometry, the incident electric field (Iω) generates a second harmonic signal (I2ω) along the parallel and crossed polarization directions. Therefore, by considering the components (α and β) of the second-order susceptibility tensor, the intensity of the SHG signals of the point group (Cs) can be expressed as . The values of each component of the susceptibility tensors obtained by fitting were α = 0.5 and β = 2.25 for 1L and α = 0.85 and β = 1.90 for 8L–9L samples. We assume that deviations may be caused by the substrate-induced strain effect or some other possible structural imperfection. 
The bilayer sample was initially analyzed by TSHG microspectroscopy because it is the simplest form of the theoretically available SHG, even under strain-free conditions. Figure 2a is a schematic energy diagram of the bilayer ReS2, which depicts the allowed energy transitions induced by the pump and probe beams. The time delay () of the probe beam is schematically illustrated; further, instantaneous two-photon absorption with a high-energy band gap (C exciton or nesting band) is known to be resonated.10, 16, 28 The time-dependent TSHG profiles for three different pump pulses (1.45, 1.57, and 1.75 eV with an ~0.6 mJ/cm2 fluence) are displayed in Figure 2b. The position of the zero probe delay (time zero) condition was determined based on the maximum sum frequency generation at 2.76 eV by the overlap of the pump and probe beams. A high signal-to-noise (S/N > 50) decay-to-rise-type TSHG profile was observed, which was caused by the loss in the probing SHG signal when the pump beam was tuned to the A excitonic position of 1.57 eV (Figure S8, Supporting Information). The sharp decay near the zero probe delay time was attributed to the electronic effect (bound charge depletion and band gap shrinkage).23, 29–31 The cross-correlation of the pump and probe beams was illustrated using a Gaussian profile with a full width at half maximum of 120 fs for comparison. Based on the literature,31 the decay dynamics of 2D material systems can be modeled using a traditional biexponential fitting or an Auger-related energy-transferring mechanism either through exciton-exciton annihilation (Rate  Ne2) or through three-body interaction (Rate  Ne3). Given a certain period of time (< 40 ps), the exciton-mediated Auger recombination model is sufficient to explain most of the time-resolved SHG spectra. Figure 2c depicts an optical image of the 2L ReS2 flake; the corresponding TSHG experiment was performed in the absence (second image) and presence (third image) of a pump beam (the dotted circle position i in Figure 2b). The TSHG signal (ΔI) and its phase information can be simply defined as the difference between the SHG signals obtained with and without the pump beams and can be given as follows.18 See the Supporting Information for the detailed derivation, Section 4b.
               ...................(2)                             
The polar plots of SHG in the absence of a pump beam and ΔI(2ω) of the bilayer sample are illustrated in Figure 2d and e. The images corresponding to various rotation angles are provided in the Supporting Information (Figure S6). The lattice constants (α and β) for the probe-only case (Figure 2d) were determined to be 0.5 and 2.42, respectively. We can clearly observe the formation of a butterfly pattern due to the gradual dipping in the middle of the lobes, which was similar to that in the case of the monolayer. The polarization-dependent TSHG (ΔI) was achieved by subtracting the probe-only SHG from the SHG of the deepest valley position, leading to the appearance of two new fitting parameters (r and k) associated with the cross-correlating electromagnetic field.18 The four parameters (α, β, r, and k) used to fit the TSHG were 0.35, 4.3, 0.7, and 0.01, respectively, even though the final term was quite negligible, which caused the elimination of the χ(2) term in the signal. The intensity decrease was most drastic at ~45° from the b axis, resulting in the formation of four-fold symmetry petals due to the dominance of the β term in Eq. (2). This χ(3)-dominant event is analogous to the case of the four-fold polarization dependence of MoS2 TSHG spectroscopy when the polarization of the beam was rotated.23 No clear evidence proving the presence of structural disordering by monitoring the polarization dependence of further probe delay conditions was noted.
The carrier dynamics of the bulk region was investigated using a 13L sample with a pump fluence of 0.6, 1.3, 3, and 8 mJ/cm2. The results are summarized in Figure 3a. A clear decay-to-rise trend near the zero probe delay, followed by a gradual recovery of the SHG response, was obtained for each fluence. The maximum power level employed to perform our study was set below the Mott transition fluence condition (~10 mJ/cm2).23, 32 The fluences applied in our experiment were high enough to produce photoexcited electrons in the range of 1012 to 1013 cm−2.21 The SHG images captured at the zero probe delay position for each of the pump powers are presented on the top right of Figure 3a (refer to Figure S8 for detailed pump fluence-dependent TSHG images, Supporting Information), confirming the damage-free conditions of the ReS2 crystals exposed to the aforementioned power levels. Further, as the power level increased, the dip near time zero further increased; the large number of carriers excited to the conduction band gradually affected the structural modulation because the injecting pump fluence was close to the level of one photon absorption per unit cell (~10 mJ/cm2).23, 29–32 Prior results illustrated that intense femtosecond laser pulses can agitate the second-order susceptibility of bulk inorganic semiconductors, such as GaAs and InSb, at a different time scale.29, 30 Additionally, the evolution of a highly populated state with a higher laser fluence (8 mJcm-2) is likely to cause a long recovery time (> 0.5 ns).23
A zoomed-in view of the TSHG profile in the inset demonstrates that all the electronic decay dynamics roughly occurred within ~0.5 ps, while the slight initial rise observed in the 8 mJ/cm2 excitation is likely related to the pseudoexcited state absorption caused by the temporally populated electrons (~1013/cm2) in the conduction band. Additionally, the carrier lifetime is inversely proportional to the input pump energy, as illustrated in the bottom right of Figure 3a. This result is in agreement with the defect-assisted Auger recombination model (dn/dt = −Rc × n × p, where n and p are the electron and hole densities, respectively; Rc is the recombination constant) when the electron and hole densities are identical.33, 34 Further, the direct band transition and the subsequent radiative electron–hole recombination are restricted to the bulk system; thus, the generated heat is more likely to be consumed in the form of phonons or Auger recombinations.18 However, the phonon-assisted carrier recombination is effectively activated when the lattice temperature is highly elevated. Therefore, the Auger recombination process will play a predominate role in relaxing the excited electrons that must have originated from the effect of the weak electron coupling of the interlayers of the ReS2 crystals in comparison with that of the multilayer MoS2 crystals. In addition, the Auger recombination process is known to be an important mechanism to capture not only free but also bound electrons. Sun et al.35 suggested a simple kinetic model (dnx/dt = −R × nx2) to explain the dynamics of the exciton–exciton annihilation in monolayered MoS2, where the solution could be represented as nx−1fluence−1R × t. Here, the exciton density (nx) is inversely proportional to the carrier lifetime (1/t) for a certain level of photoexcitation. The parameter R represents the recombination rate constant (~0.7 cm2/s), which is approximately one order of magnitude larger than that of the MoS2 monolayers (Figure S9 in Section 5, Supporting Information). The defect states of the bulk ReS2 systems were also investigated using laser power-dependent PL emission (λex = 520 nm), which confirmed the presence of defect state emission and the band edge exciton, and the anomalous broadening of the PL spectra obtained at a high excitation power indicated the influence of the defect states (Supporting Information, Figure S7 and S10).36
Subsequently, the variation in TSHG spectra from 2- to 16-layer samples was studied (Figure 3b). Optical and TSHG images representative of the different delay positions are illustrated on the top right (Figure S11, Supporting Information). Sharp decays before time zero were noted again, within a period of ∼2 ps (I), regardless of the sample thickness in the case of an identical (~1 mJcm-2) pump fluence. In this short temporal range, the SHG recovery could be significantly influenced by electron–electron scattering, defect ionization, and/or the thermalization of the photoexcited carrier by optical phonon emission.21, 33,34 For all sample thicknesses, an inflection in the TSHG spectra (change in the rapid-to-slow recovery trend) was observed between 2 and 5 ps of probe delay (II). The region labeled “III” exhibited a gradual damping of the electron recovery process as a function of the increasing layer thickness, indicating the prominent feature of a slow three-body Auger recombination process within the bulk system. We fitted the observed transient features of the TSHG curves with the second exponential terms after the inflecting points (red solid lines). The fitted lifetimes and layer thicknesses are summarized at the bottom right: 2L, 68; 3L, 88; 5L, 106; 9L, 113; 13L, 124; and 16L, 135 ps (refer to Figure S12 for details about the biexponential fitting, Supporting Information). The observed time scales demonstrate an interesting relation with the layer number, similar to that observed for the MoS2 layers, indicating that the carrier lifetime variation is caused by the combination of the surface and bulk Auger-related recombination. To balance the surface and bulk contributions, we additionally assumed a model based on the probability density of electrons, similar to that in the MoS2 case33, 34 (details are provided in Section 6 of the Supporting Information). The values calculated for  matched the experimentally observed lifetimes, as depicted at the bottom right of Figure 3b, which indicated that the lifetime of the carrier was strongly controlled by the defect states of the ReS2 crystal, as previously reported for MoS2, except for a slight appearance of the long three-body Auger recombination process (1 ns level in MoS2).
In the following section, we discuss the mechanisms of the electronic transitions observed in the TSHG spectra of the bilayer ReS2 crystals. As discussed above, the usage of a pump energy under excitonic resonance conditions (1.57 eV) and a probe energy (1.19 eV) targeted to 2.38 eV caused the formation of a decay-to-rise TSHG profile. This tendency is opposite to that observed in the case of an MoS2 crystal measured under corresponding excitonic resonance conditions (near 1.8 eV).18 To understand the different electronic transitions, in addition to the aforementioned ones, we employed a new wavelength-fixed pump (2.38 eV) associated with tunable probe beams (1.3 to 1.41 eV). The extracted TSHG spectra were normalized and organized to better understand Figure 4a; the corresponding optical and SHG images are illustrated at the top. A modulation in the probe energy clearly affirmed the distinct change in the trend from decay-to-rise (1.30–1.34 eV) to rise-to-decay (1.37–1.41 eV). Those change are not simply due to the probe energy dependent anisotropic TSHG as we confirmed by measuring the angle dependent TSHG for the two different (1.3 ev and 1.41 eV) probe condition (refer to the Figure S13, Supporting Information). A similar change in the rise-to-decay profile to a decay-to-rise profile was observed in the TSHG spectrum of the monolayer MoS2, which seems to be related to the existence of an energy state that allowed for excited state absorption (ESA) at 6.02 eV from the HOMO state; the states vanished at 7.76 eV.23 However, the recovery time (stage II) of the rise-to-decay profile was approximately doubled compared with that of the decay-to-rise profile reported in the literature, which is in contrast to the trend observed in our study. The competition between GSD and ESA may be slightly biased towards GSD with 1.30 eV (at 4.98 eV), which allows for bleaching of the ground state with a limited efficiency. Additionally, the gradual appearance of the rise-to-decay trend in the early stage of the TSHG profile with a 1.34-eV probe (at 5.04 eV) implied that the competition between GSD and ESA is time dependent, where the initiated GSD switched to ESA for 1 ps, followed by a sudden return. Further, to specify the ground state absorption of the probe pulses within the electronic bands of the ReS2 crystal, we measured the reflection spectra of 2L ReS2, as depicted in Figure 4b. The spectra reveal that the pump beam at 2.38 eV can be strongly absorbed, whereas the linear absorptions of the probe beams are negligible, irrespective of the allowance of their absorption induced by the two-photon action in the 2ω region. Based on the experimental absorption data and postulates regarding the behavior of the SHG probe reported in the literature, we suggest two schemes that can distinguish between the decay-to-rise and rise-to-decay profiles in Figure 4c. We assume that the pump beam used up ground state electrons assigned for generating SHG by 1.30 and 1.34 eV probe beams, leading to the decay-to-rise profile. Further, an additional ESA is allowed in the cases that exhibited the probing of 1.37 and 1.41 eV due to the presence of a certain transition-permitted energy state. Note that the ESA corresponds to an “increment” in SHG due to the additional signal of the probe beams induced by ESA in addition to the intrinsic SHG without the pump source.
Along with the GSD domination in the TSHG profile when combining the 1.57-eV pump with the 1.19-eV probe, the alternating appearances of ESA and GSD with a probe beam difference of only a few tenths of meV should be verified. The allowance of an electronic transition could be directly determined using the angular momentum conservation rule (ΔL = ±1). However, unlike populated homogeneous molecules with fixed eigenstates, the highly degenerate electron structures of 2D materials prevent the precise elucidation of the selectivity of their electron transitions. Therefore, we initially performed density functional theory (DFT) calculations, implemented using the VASP code, to determine the availability of quantum states for each pump and probe absorption. The band structures theoretically calculated for 2L ReS2 are depicted in Figure 5a (refer to the Supporting Information for calculation details). The results exhibit that the minimum direct band gap is ~1.40 eV at the Γ point, which matches the electronically measured and theoretically calculated values reported in the literature.8, 9 In fact, this value is somewhat lower than the excitonic resonance (1.57 eV) optically measured for the 2L ReS2 crystal and can be adjusted by a compressive strain of ~2%, reaching 1.53 eV, an ~2.5% difference from 1.57 eV with the upper band structure intact. Notably, the calculation predicts that there is an additional small-size band gap (~0.5 eV) above the band gap at the Γ point (4.55–5.05 eV from the HOMO). Given the 1.57-eV pump, the interband transition of electrons was seemingly dominated by the p-d transitions. The ESA behavior was not experimentally permitted, although many degenerate electron states existed above the bottom edge of the conduction band by as much as 2.38 eV (i.e., 3.95 eV above the ground state), as summarized in Figure 5a. To explain this phenomenon, we categorized the degenerate conduction band states according to the subshell orbital types (Figure 5b and 5c), among which most comprised Re d and s and S p orbitals. Note that the populated electrons at the bottom edge of the conduction band excited by a pump energy of 1.57 eV have a limited probability of being probed by SHG because the involved electronic transition is triggered by instantaneous two-photon absorption via the d–d transition path, which is forbidden based on the selection rule (ΔL = 0). More specifically, the dominance of the d orbital (~70%) at the bottom edge position is retained (~65%) at the electronic transition level of the second probe absorption (3.95 eV from the ground state), which further limits the appearance of ESA. Additionally, a 1-eV gap at the Γ position is another factor that restricts the fast electronic transition without the assistance of the optical phonon. Once the electrons are excited in the case of a 2.38-eV pump, the two-photon absorption by the 1.41-eV probe photon will successfully occur based on ΔL = 1 in the transition from d (L = 2) to the hybrid orbital of Re s and S p (5.10 eV from the HOMO band) because the hybrid orbital is characterized by the antibonding of Re s and S p and the L = 1 channel dominates the angular momentum expansion due to its antisymmetry.
 			(3)
    A schematic based on the aforementioned calculation is shown (Figure 5d) to better understand the hybridization of the Re s and S p orbitals and the related electronic density of the states that comprised the Re s and S p and Re d orbitals. However, the same two-photon action is unlikely to occur when using the 1.30-eV probe because the position located at a distance of 4.98 eV from the HOMO band is located in the upper band gap. Note that the Re s orbitals at ~5.10 eV are highly populated compared with the Mo s orbital density in the case of the MoS2 system based on our calculation, where the direct band gap is located at K (data not shown). 


Discussion 
We demonstrated the possible existence of a 0.5-eV band gap at a distance of 4 eV from the ground state of the bilayer ReS2 crystal using time-resolved TSHG microscopy. We initially investigated the lower part of the gap using a 1.19-eV probe (2.38 eV SHG) and wavelength-variable pump photons from 1.41 to 1.72 eV. Here, the probe-only thickness-dependent SHG gradually plateaued in the ~13-nm layer and was closely modeled using the dipole summation and self-interferometric absorption mechanisms; the polarization dependence of the SHG varied in a layer-by-layer manner, following the model designed for the distorted 1T crystal. Each layer exhibited maximized GSD behavior in the presence of the 1.57-eV pump beam (corresponding to the nondegenerate direct band gap), with excitonic lifetimes that were typically modeled as Auger-assisted surface-dominant electron–hole recombination. Time-dependent rise-to-decay behavior (i.e., the ESA condition) was not observed because the two-photon transition (2.38 eV) that followed band gap pumping (1.57 eV) corresponded to the forbidden intraconduction band d–d transition. Finally, the usage of a frequency-doubled pump (2.38 eV) and various probe energies (1.37–1.41 eV) enabled us to perform analysis at a distance up to 5.2 eV from the ground state, thereby revealing a new allowed ESA at ~5.05 eV. The modified VASP-type DFT calculations revealed that this transition is strongly related to the “allowed d–s transition” and further confirmed the existence of another gap with a size of ~0.5 eV. Our microscopic visualization based on the thickness-dependent modeling of the SHG, spatiotemporal assessment of carrier dynamics, and confirmation of the existence of a higher energy band gap of ReS2 crystalline systems ascertained the benefit of TSHG microscopy for understanding the fundamental physical properties required to design new optoelectronic devices based on TMD and van der Waals-related heterostructures.

Materials and methods
Sample preparation and optical characterization 
Single crystals of ReS2 with various layer thicknesses were exfoliated on a Si/SiO2 substrate from a commercially available natural crystal (2D semiconductor company). These crystals were directly used to study the Raman, PL, absorption and nonlinear optical properties at room temperature. Details of the instrumentation for the optical properties and correlated electronic band structure are provided in Supporting Information Section 1.

Theoretical calculation 
We employed the first-principles electronic structure calculation performed on the Vienna ab initio simulation package (VASP) code with the Perdew-Burke-Ernzerhof (PBE) generalized gradient approximation (GGA) exchange correlation functional37-39 and a Monkhorst-Pack40 grid of a 9×9×1 k-points mesh. An energy cutoff of 500 eV was adopted. The freestanding bilayer 1T’ ReS2 was calculated with Dudarev’s DFT+U41 correction of 5 eV for the p orbitals of S atoms.

Acknowledgements
This work was supported by the National Research Foundation of Korea (2015R1A3A2066337, 2017K1A3A1A19070455, 2016R1A2B4013141) and the basic research programs (18-LC-01) through the Daegu Gyeong-buk Institute of Science and Technology (DGIST) funded by the Ministry of Science and ICT of Korea. 
Author Details
1School of Electrical and Electronic Engineering, Yonsei University, Seoul 03722, Republic of Korea. 2Companion Diagnostics & Medical Technology Research Group, DGIST, Daegu 42988, Republic of Korea. 3Department of Emerging Materials Science, DGIST, Daegu 42988, Republic of Korea.

Author Information
These authors contributed equally: Krishna P. Dhakal, Hyunmin Kim
Author Contributions
H.K., J.D.L. and J.-H.A planned and supervised the project. K.P.D. prepared all the samples. K.P.D. and H.K. conducted all experiments; S.L. and Y.K. performed the theoretical calculations. K.P.D., J.-H.A, H.K. and J.D.L. analyzed the data and wrote the manuscript.

Conflict of Interest
The authors declare no conflict of interest

Corresponding authors: Correspondence to hyunmin.kim@dgist.ac.kr, jdlee@dgist.ac.kr and ahnj@yonsei.ac.kr

Supplementary information 
Supplementary Information for this article can be found on the Light: Science & Applications’ website (http://www.nature.com/lsa). 


References
Tongay S, Sahin H, Ko C, Luce A, Fan W et al. Monolayer behaviour in bulk ReS2 due to electronic and vibrational decoupling. Nat Commun 2014; 5: 3252. 
Chenet DA, Aslan OB, Huang PY, Fan C, van der Zande AM et al. In-plane anisotropy in mono- and few-layer ReS2 probed by raman spectroscopy and scanning transmission electron microscopy. Nano Lett 2015; 15: 5667-5672. 
Feng YQ, Zhou W, Wang YJ, Zhou J, Liu EF et al. Raman vibrational spectra of bulk to monolayer ReS2 with lower symmetry. Phys Rev B 2015; 92: 054110. 
Aslan OB, Chenet DA, van der Zande AM, Hone JC, Heinz TF. Linearly polarized excitons in single- and few-layer ReS2 crystals. ACS Photonics 2016; 3: 96-101. 
He R, Yan JA, Yin ZY, Ye ZP, Ye GH et al. Coupling and stacking order of ReS2 atomic layers revealed by ultralow-frequency raman spectroscopy. Nano Lett 2016; 16: 1404-1409. 
Qiao XF, Wu JB, Zhou LW, Qiao JS, Shi W et al. Polytypism and unexpected strong interlayer coupling in two-dimensional layered ReS2. Nanoscale 2016; 8: 8324-8332. 
Cui QN, Muniz RA, Sipe JE, Zhao H. Strong and anisotropic third-harmonic generation in monolayer and multilayer ReS2. Phy Rev B 2017; 95: 165406. 
Yu ZG, Cai YQ, Zhang YW. Robust direct bandgap characteristics of one- and two-dimensional ReS2. Sci Rep 2015; 5: 13783. 
Park JY, Joe HE, Yoon HS, Yoo SH, Kim T et al. Contact effect of ReS2/metal interface. ACS Appl Mater Interfaces 2017; 9: 26325-26332. 
Dhakal KP, Duong DL, Lee J, Nam H, Kim M et al. Confocal absorption spectral imaging of MoS2: optical transitions depending on the atomic thickness of intrinsic and chemically doped MoS2. Nanoscale 2014; 6: 13028-13035. 
Dhakal KP, Roy S, Jang H, Chen X, Yun WS et al. Local strain induced band gap modulation and photoluminescence enhancement of multilayer transition metal dichalcogenides. Chem Mater 2017; 29: 5124-5133. 
Shinde SM, Dhakal KP, Chen X, Yun WS, Lee JD et al. Stacking-controllable interlayer coupling and symmetric configuration of multilayered MoS2. NPG Asia Mater 2018; 10: e468. 
Li YL, Rao Y, Mak KF, You YM, Wang SY et al. Probing symmetry properties of few-layer MoS2 and h-BN by optical second-harmonic generation. Nano Lett 2013; 13: 3329-3333. 
Suzuki R, Sakano M, Zhang YJ, Akashi R, Morikawa D et al. Valley-dependent spin polarization in bulk MoS2 with broken inversion symmetry. Nat Nanotechnol 2014; 9: 611-617. 
Jiang T, Liu HR, Huang D, Zhang S, Li YG et al. Valley and band structure engineering of folded MoS2 bilayers. Nat Nanotechnol 2014; 9: 825-829. 
Malard LM, Alencar TV, Barboza APM, Mak KF, de Paula AM. Observation of intense second harmonic generation from MoS2 atomic crystals. Phy Rev B 2013; 87: 201401. 
Zhao M, Ye ZL, Suzuki R, Ye Y, Zhu HY et al. Atomically phase-matched second-harmonic generation in a 2D crystal. Light: Sci Appl 2016; 5: e16131. 
Jang H, Dhakal KP, Joo KI, Yun WS, Shinde SM et al. Transient SHG imaging on ultrafast carrier dynamics of MoS2 nanosheets. Adv Mater 2018; 30: 1705190. 
Mak KF, He KL, Shan J, Heinz TF. Control of valley polarization in monolayer MoS2 by optical helicity. Nat Nanotechnol 2012; 7: 494-498. 
Gehlmann M, Aguilera I, Bihlmayer G, Nemšák S, Nagler P et al. Direct observation of the band gap transition in atomically thin ReS2. Nano Lett 2017; 17: 5187-5192. 
Cui QN, He JQ, Bellus MZ, Mirzokarimov M, Hofmann T et al. Transient absorption measurements on anisotropic monolayer ReS2. Small 2015; 11: 5565-5571. 
Sim S, Lee D, Noh M, Cha S, Soh CH et al. Selectively tunable optical Stark effect of anisotropic excitons in atomically thin ReS2. Nat Commun 2016; 7: 13569. 
Mannebach EM, Duerloo KAN, Pellouchoud LA, Sher MJ, Nah S et al. Ultrafast electronic and structural response of monolayer MoS2 under intense photoexcitation conditions. ACS Nano 2014; 8: 10734-10742. 
Dyakonov MI. Spin Physics in Semiconductors. Berlin, Heidelberg: Springer; 2008. p379. 
Beams R, Cançado LG, Krylyuk S, Kalish I, Kalanyan B et al. Characterization of few-layer 1T′ MoTe2 by polarization-resolved second harmonic generation and raman scattering. ACS Nano 2016; 10: 9626-9636. 
Cui FF, Wang C, Li XB, Wang G, Liu KQ et al. Tellurium-assisted epitaxial growth of large-area, highly crystalline ReS2 atomic layers on mica substrate. Adv Mater 2016; 28: 5019-5024. 
Lin YC, Komsa HP, Yeh CH, Björkman T, Liang ZY et al. Single-layer ReS2: two-dimensional semiconductor with tunable in-plane anisotropy. ACS Nano 2015; 9: 11249-11257. 
Kozawa D, Kumar R, Carvalho A, Amara KK, Zhao WJ et al. Photocarrier relaxation pathway in two-dimensional semiconducting transition metal dichalcogenides. Nat Commun 2014; 5: 4543. 
Glezer EN, Siegal Y, Huang L, Mazur E, Behavior of χ(2) during a laser-induced phase transition in GaAS. Phy Rev B 1995; 51: 9589-9596. 
Shumay IL, Höfer U. Phase transformations of an InSb surface induced by strong femtosecond laser pulses. Phy Rev B 1996; 53: 15878-15884. 
Trinh MT, Wu XX, Niesner D, Zhu XY. Many-body interactions in photo-excited lead iodide perovskite. J Mater Chem A 2015; 3: 9285-9290. 
Chernikov A, Ruppert C, Hill HM, Rigosi AF, Heinz TF. Population inversion and giant bandgap renormalization in atomically thin WS2 layers. Nat Photon 2015; 9: 466. 
Wang HN, Zhang CJ, Rana F. Ultrafast dynamics of defect-assisted electron-Hole recombination in monolayer MoS2. Nano Lett 2015; 15: 339-345. 
Wang HN, Zhang CJ, Rana F. Surface recombination limited lifetimes of photoexcited carriers in few-layer transition metal dichalcogenide MoS2. Nano Lett 2015; 15: 8204-8210. 
Sun DZ, Rao Y, Reider GA, Chen GG, You YM et al. Observation of rapid exciton-exciton annihilation in monolayer molybdenum disulfide. Nano Lett 2014; 14: 5625-5629. 
Tongay S, Suh J, Ataca C, Fan W, Luce A et al. Defects activated photoluminescence in two-dimensional semiconductors: interplay between bound, charged, and free excitons. Sci Rep 2013; 3: 2657. 
Kresse G, Furthmüller J. Efficiency of ab-initio total energy calculations for metals and semiconductors using a plane-wave basis set. Comput Mater Sci 1996; 6: 15-50. 
Perdew JP, Burke K, Ernzerhof M. Generalized gradient approximation made simple. Phys Rev Lett 1996; 77: 3865-3868. 
Kohn W, Sham LJ. Self-consistent equations including exchange and correlation effects. Phys Rev 1965; 140: A1133-A1138. 
Monkhorst HJ, Pack JD. Special points for Brillouin-zone integrations. Phys Rev B 1976; 13: 5188-5192. 
Dudarev SL, Botton GA, Savrasov SY, Humphreys CJ, Sutton AP. Electron-energy-loss spectra and the structural stability of nickel oxide: an LSDA+ U study. Phys Rev B 1998; 57: 1505-1509. 


Figure legends
Figure 1. Layer number and polarization-dependent SHG intensity variation. a) Diagram of the time-resolved second harmonic generation (TSHG) microscopy and sample configuration used in this study. A top view schematic of the 1L lattice is presented on the left. b) SHG micrographs of the region with various layer thicknesses (1L, 2L, and 3L to bulk crystals). The scale bar corresponds to 2 μm. The corresponding optical images are depicted in the inset of the graph. The graph represents the measured (black circle) and calculated (red circle) layer thickness-dependent SHG intensity of the ReS2 crystals. c) Optical images of 1L and multilayer (8L–9L) ReS2 crystals. The scale bar corresponds to 10 μm. The polarization-dependent SHG intensity (1.19 eV) variations of the 1L d) and multilayer e) samples were obtained by rotating the sample. The data are normalized and plotted. The red line represents the fit of the angle-dependent SHG variation. The polarization of the analyzer is parallel to the beam polarization direction (double-sided arrow).

Figure 2. Transient SHG of the ReS2 crystal. a) A schematic of the overlapped probe and pump beams for an energy band structure of bilayer ReS2. b) The pump beams with three different energies (1.72, 1.57, and 1.45 eV) tuned to the excitonic resonance were overlapped with the probe beam to create a time-dependent SHG response as a function of the probe delay. c) Optical and SHG images of the 2L ReS2 crystal in the absence and presence of the pump beam. The double-sided arrow depicts the direction of the beam polarization; the scale bar indicates 5 μm. d) Polarization-dependent SHG intensity without the pump; e) increment with the pump from (d). The magenta lines represent the fit of the angle-dependent SHG variations.

Figure 3. Pump power and layer number-dependent transient SHG spectra. a) TSHG spectra of the 13-nm ReS2 crystal obtained at different pump fluences (0.6, 1.3, 3, and 8 mJ/cm2). The zoomed-in view of the dip near a zero probe delay (time zero) is illustrated in the inset. The respective SHG images are displayed on the top right. The scale bars correspond to 2 μm. The observed inverse carrier lifetime “1/τ” (bottom right) increases linearly with increasing pump fluence. b) Normalized TSHG of the 2L to 16L ReS2 crystals obtained with the 1.57-eV pump and 1.19-eV probe beams. c) Representative SHG images captured at different probe delay times. The carrier lifetime (τ) as a function of the number of layers is plotted at the bottom right. The uncertainties of the extracted lifetime are also projected, including error bars. All the scale bars correspond to 5 μm. 

Figure 4. Experimental evidence of the probe wavelength-dependent tunable decay profile of the ReS2 crystal. a) TSHG spectra of the 2L crystal as a function of the probe energy from 1.3 to 1.41 eV when the pump energy was maintained at 2.38 eV. Representative optical and SHG images of the sample are given in the top panel. The scale bar corresponds to 3 μm. b) Optical reflectance spectra of the 2L ReS2 crystal. The relative positions of the applied pump, probes, and resulting SHG energies are marked in the spectrum. c) Illustrations of the electronic transitions corresponding to the decay-to-rise and rise-to-decay profiles.

Figure 5. Theoretical calculation for the interaction among molecular orbitals and density of states for the ReS2 crystal. a) Electronic structure of the 2L ReS2 crystal calculated using the VASP DFT method after being pseudocolor-coded according to the Re d orbital (red) and the Re s (blue) and S p (green) orbitals. The presence of the lower (1.40 eV) and upper (0.5 eV) band gaps is demonstrated. The relative weight b) between the Re d and S p orbitals and c) between the Re d, Re s, and S p orbitals. The abbreviations GSD and ESA stand for ground state depletion and excited state absorption, respectively. d) Illustration of the hybrid wavefunction of the Re s and S p orbitals and the associated density of states of the involved orbitals (Re s, Re d, and S p). 


Figure 1. Layer number and polarization-dependent SHG intensity variation. a) Diagram of the time-resolved second harmonic generation (TSHG) microscopy and sample configuration used in this study. A top view schematic of the 1L lattice is presented on the left. b) SHG micrographs of the region with various layer thicknesses (1L, 2L, and 3L to bulk crystals). The scale bar corresponds to 2 μm. The corresponding optical images are depicted in the inset of the graph. The graph represents the measured (black circle) and calculated (red circle) layer thickness-dependent SHG intensity of the ReS2 crystals. c) Optical images of 1L and multilayer (8L–9L) ReS2 crystals. The scale bar corresponds to 10 μm. The polarization-dependent SHG intensity (1.19 eV) variations of the 1L d) and multilayer e) samples were obtained by rotating the sample. The data are normalized and plotted. The red line represents the fit of the angle-dependent SHG variation. The polarization of the analyzer is parallel to the beam polarization direction (double-sided arrow).


Figure 2. Transient SHG of the ReS2 crystal. a) A schematic of the overlapped probe and pump beams for an energy band structure of bilayer ReS2. b) The pump beams with three different energies (1.72, 1.57, and 1.45 eV) tuned to the excitonic resonance were overlapped with the probe beam to create a time-dependent SHG response as a function of the probe delay. c) Optical and SHG images of the 2L ReS2 crystal in the absence and presence of the pump beam. The double-sided arrow depicts the direction of the beam polarization; the scale bar indicates 5 μm. d) Polarization-dependent SHG intensity without the pump; e) increment with the pump from (d). The magenta lines represent the fit of the angle-dependent SHG variations.


Figure 3. Pump power and layer number-dependent transient SHG spectra. a) TSHG spectra of the 13-nm ReS2 crystal obtained at different pump fluences (0.6, 1.3, 3, and 8 mJ/cm2). The zoomed-in view of the dip near a zero probe delay (time zero) is illustrated in the inset. The respective SHG images are displayed on the top right. The scale bars correspond to 2 μm. The observed inverse carrier lifetime “1/τ” (bottom right) increases linearly with increasing pump fluence. b) Normalized TSHG of the 2L to 16L ReS2 crystals obtained with the 1.57-eV pump and 1.19-eV probe beams. c) Representative SHG images captured at different probe delay times. The carrier lifetime (τ) as a function of the number of layers is plotted at the bottom right. The uncertainties of the extracted lifetime are also projected, including error bars. All the scale bars correspond to 5 μm. 


Figure 4. Experimental evidence of the probe wavelength-dependent tunable decay profile of the ReS2 crystal. a) TSHG spectra of the 2L crystal as a function of the probe energy from 1.3 to 1.41 eV when the pump energy was maintained at 2.38 eV. Representative optical and SHG images of the sample are given in the top panel. The scale bar corresponds to 3 μm. b) Optical reflectance spectra of the 2L ReS2 crystal. The relative positions of the applied pump, probes, and resulting SHG energies are marked in the spectrum. c) Illustrations of the electronic transitions corresponding to the decay-to-rise and rise-to-decay profiles.


Figure 5. Theoretical calculation for the interaction among molecular orbitals and density of states for the ReS2 crystal. a) Electronic structure of the 2L ReS2 crystal calculated using the VASP DFT method after being pseudocolor-coded according to the Re d orbital (red) and the Re s (blue) and S p (green) orbitals. The presence of the lower (1.40 eV) and upper (0.5 eV) band gaps is demonstrated. The relative weight b) between the Re d and S p orbitals and c) between the Re d, Re s, and S p orbitals. The abbreviations GSD and ESA stand for ground state depletion and excited state absorption, respectively. d) Illustration of the hybrid wavefunction of the Re s and S p orbitals and the associated density of states of the involved orbitals (Re s, Re d, and S p). 
