# Supplementary figures and images for: Probing the upper band gap of atomic rhenium disulfide layers
Source: Light Sci Appl. 2018 Nov 28;7:98. doi: 10.1038/s41377-018-0100-3 (PMC6262017; doi:10.1038/s41377-018-0100-3)

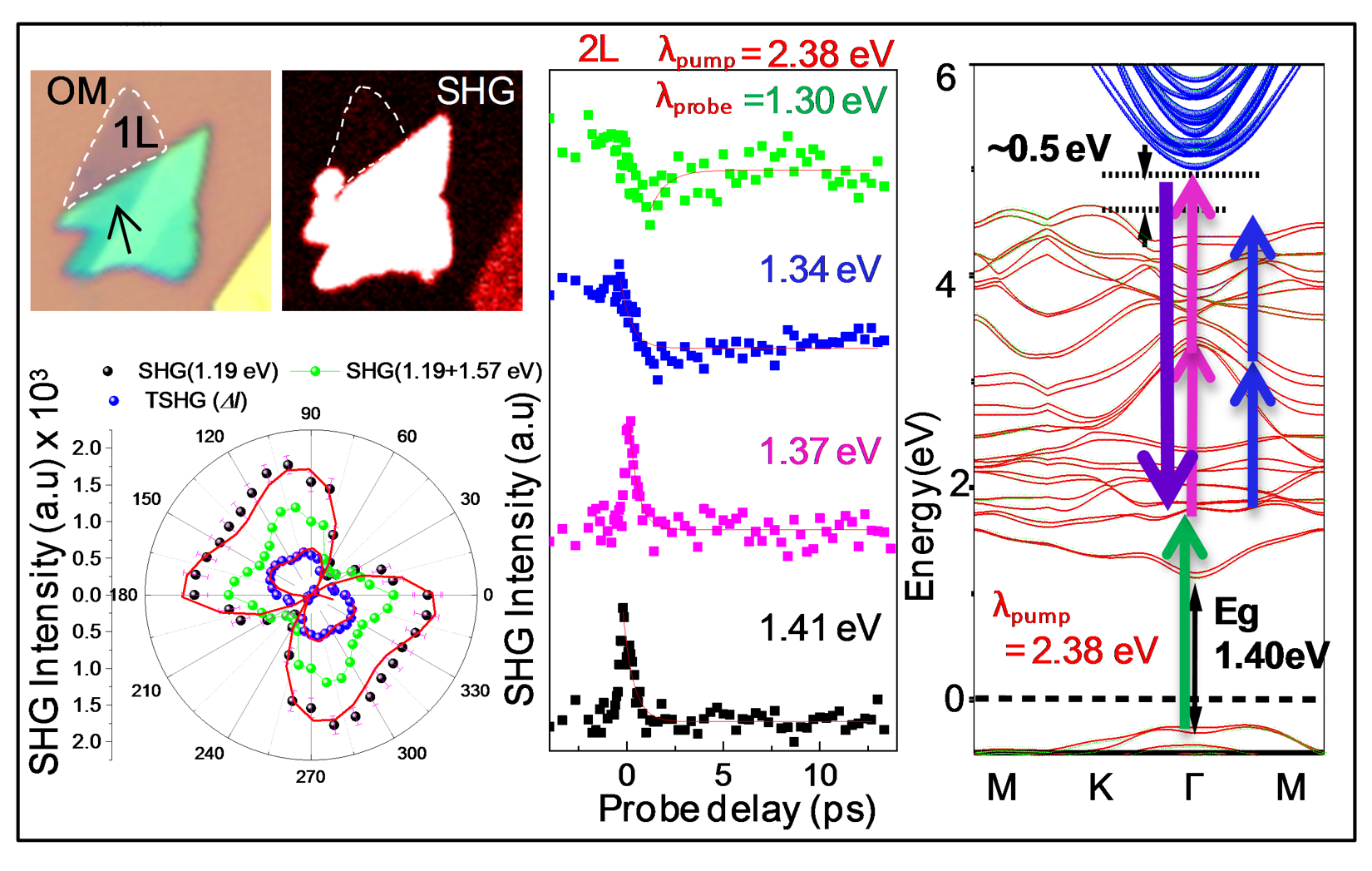

Supplement: Supplementary file 3 — table of content [file 41377_2018_100_MOESM3_ESM.tif]
